# SP/NK‐1R promotes gallbladder cancer cell proliferation and migration

**DOI:** 10.1111/jcmm.14230

**Published:** 2019-03-22

**Authors:** Xue‐Ting Deng, Si‐Min Tang, Pei‐Yao Wu, Quan‐Peng Li, Xian‐Xiu Ge, Bo‐Ming Xu, Hui‐Shan Wang, Lin Miao

**Affiliations:** ^1^ Medical Center for Digestive Diseases the Second Affiliated Hospital of Nanjing Medical University Nanjing China

**Keywords:** Akt, gallbladder cancer, NF‐κB, NK‑1R, substance P

## Abstract

Aberrant substance P/neurokinin‐1 receptor (SP/NK‐1R) system activation plays a critical role in various disorders, however, little is known about the expression and the detailed molecular mechanism of the SP and NK‐1R in gallbladder cancer (GBC). In this study, we firstly analyzed the expression and clinical significance of them in patients with GBC. Then, cellular assays were performed to clarify their biological role in GBC cells. Moreover, we investigated the molecular mechanisms regulated by SP/NK‐1R. Meanwhile, mice xenografted with human GBC cells were analyzed regarding the effects of SP/NK1R complex in vivo. Finally, patient samples were utilized to investigate the effect of SP/NK‐1R. The results showed that SP and NK‐1R were highly expressed in GBC. We found that SP strongly induced GBC cell proliferation, clone formation, migration and invasion, whereas antagonizing NK‐1R resulted in the opposite effects. Moreover, SP significantly enhanced the expression of NF‐κB p65 and the tumor‐associated cytokines, while, Akt inhibitor could reverse these effects. Further studies indicated that decreasing activation of NF‐κB or Akt diminished GBC cell proliferation and migration. In consistent with results, immunohistochemical staining showed high levels of Akt, NF‐κB and cytokines in tumor tissues. Most importantly, the similar conclusion was obtained in xenograft mouse model. Our findings demonstrate that NK‐1R, after binding with the endogenous agonist SP, could induce GBC cell migration and spreading via modulation of Akt/NF‐κB pathway.

## 1 INTRODUCTION

Gallbladder cancer (GBC) is a rare and high lethal cancer that differs from others involving the gastrointestinal tract, as being three times more common in women than men.^1^ This tumor accounts for 80%‐95% of biliary tract malignancies and has an extremely dismal prognosis.^2^ Although great progress has been made in the treatment of GBC with the use of radical surgical approach, the presenting symptoms of patients are not specific so that the diagnosis commonly occurs at an advanced stage.^3^ Therefore, the survival percentage of patients diagnosed to have gallbladder cancer is less than 5% in 5 years.^4^ For this reason, a better understanding of genomic and molecular profiles involved in GBC development and progression would consider as the novel strategies to improve the survival of GBC patients.

Neurokinin‐1 receptor (NK‐1R) is a tachykinin receptors which functionally coupled with G‐protein and then following by the involvement in human biological responses.^5^ Recent articles indicate NK‐1R is widespread distribution in the mammalian central nervous system and peripheral tissues. Most importantly, NK‐1R regulates many biological functions in neurogenic inflammation, immune responses, pain and depression. And it has also been implicated to play a vital role in regulating numerous pathophysiological actions related to cancer.^6^, ^7^, ^8^, ^9^ After combining specifically with the SP, one of the tachykinins, NK‐1R controls the tumor cell proliferation, migration, metastasis and angiogenesis.^6^, ^7^, ^10^ However, NK‐1R antagonists could block the function of SP/NK‐1R complex and induce apoptosis and death of cancer cells. Studies by J. Zhang et al. and M. Munoz et al. indicate an important role of SP/NK‐1R signaling in cancer progression and metastasis, as well as tumor microenvironment.^11^, ^12^ Furthermore, it has been reported that SP/NK‐1R complex potently modulates several signaling cues including Wnt, ERK and Akt in many primary and metastatic human cancer.^13^, ^14^, ^15^ Amongst these signalings, it is imperative to know that Akt has been demonstrated to mediate various pathways of metabolism, proliferation and survival in tumorigenesis, and it is hyperactivated in GBC.^16^, ^17^ For instance, Y. Liu et al. report that activation of the Akt signaling pathway could help to increase GBC cell proliferation, migration and invasion.^18^ Moreover, Z. Hu et al. also find that the phosphorylation of Akt could be regulated to promote GBC cell growth, metastasis and epithelial‐mesenchymal transition (EMT).^19^ In additionally, recent evidences indicate the occurrence of Akt hyperactivation can significantly influence its downstream effectors, such as NF‐κB, which mediates cell survival and proliferation.^20^, ^21^ Researchers find that Akt transiently binds to the inhibitor of kappa B kinase (IKKα) and induces IKK activation, which in turn allows NF‐κB to translocate to the nucleus and trigger expression of target genes.^22^ Furthermore, it is found that inactivation of Akt could prevent the progression of breast cancer by block the NF‐κB pathway.^23^ In another study, Akt/NF‐κB signaling is important for the oncogenic effect of cell cycle regulating genes in esophageal squamous cell carcinoma tissue.^24^ However, the expression and functional role of SP/NK‐1R in GBC remain unknown.

Presently, nothing is known about the role of SP and NK‐1R in human GBC. In this study, we provided evidences in vitro and in vivo that the activation of NK‐1R could promote the tumorigenesis through the Akt/NF‐κB axis, and this can be reversed by NK‐1R antagonist. Furthermore, the activity of Akt, NF‐κB p65 and the other cytokines were dramatically increased in tumor tissues. Finally, animal experiments further confirmed the effects of SP/NK‐1R complex and the underlying mechanism. Taken together, we examined the effects of SP/NK‐1R and explored the potential therapeutic function on human GBC.

## 2 MATERIALS AND METHODS

### 2.1 Patients and tissues

Gallbladder cancer tissue samples were collected from patients with carcinoma of the gallbladder after informed consent and following tumor resection from the second affiliated hospital of Nanjing Medical University. Additionally, gallbladder tissue samples which collected from patients with chronic cholecystitis after informed consent and following cholecystectomy were included as controls. All work presented here was approved by second affiliated hospital of Nanjing Medical University Review Board.

### 2.2 Cell culture

Human gallbladder cancer cell lines GBC‐SD and NOZ cells were maintained in RPMI 1640 medium, supplemented with 10% fetal bovine serum (FBS) (Gibco, NY, USA). All cell lines were incubated at 37°C with 5% CO_2_.

### 2.3 Clone formation assay

Cells were plated in a 6‐well culture plate with different treatment. Triplicate wells were performed for each cell group. Plates were further cultured in an incubator until colonies were large enough to be visualized. Then, the cells were stained with 0.5% crystal violet. Afterwards, the clones with ≥50 cells were scored under the microscope.

### 2.4 RNA extraction and real‐time PCR

As described by L. Miao et al.^25^ Total RNA was extracted from cells using a RNA Rapid Extraction Kit (BioTeke Corporation, Peking, China) and reversely transcribed using a reverse transcription kit (Thermo Fisher Scientific, MA, USA) according to the manufacturer's instructions. The subsequent Real‐time PCR was performed using the SYBR^®^ Green Master Mix (Bio‐Rad Laboratories, CA, USA). Differential expression was calculated according to the 2^−ΔΔCT^ method and statistically evaluated.

### 2.5 Western blotting

As described by L. Miao et al.^25^ Total proteins were isolated from the cell lines. Protein lysates were run on SDS PAGE gels and then moved to PVDF membrane. The membrane was incubated in 3% bovine serum albumin (BSA) and then blotted with specific primary antibody. After incubation in the HRP‐labeled secondary antibody, the membrane was detected with chemiluminescence western blot detection system (Millipore, Billerica, MA, USA).

### 2.6 ELISA assay

Cell supernatants were collected after treatment with reagents. TNF‐α, IL‐1β, IL‐6 and MMP9 were measured by standardized ELISA assay (Boster, Wuhan, China) according to the manufacturer's instructions. And the protein levels of cytokines were expressed in pg/mL.

### 2.7 Wound healing assay

Cells were cultured in six‐well plates. An incision was made by a sterile 10 μL pipette tip in the central area of cell layers. After washed with PBS twice, the cells were cultured with conditioned media. The wounded gaps were captured by microscope at different time points. Migration assays were performed in triplicate.

### 2.8 Cell invasion assay

Cell transwell migration and matrigel invasion assays were performed using 24‐well transwell chambers according to the manufacturer's instructions. The upper surface of the transwell membrane was pre‐coated with Matrigel matrix (BD,USA), and the lower chamber was filled with 500 μL of culture media containing 1% FBS. Cells were seeded to the upper side in serum‐free medium for indicated time periods. Then the migrated cells on the bottom surface were fixed with ice‐cold methanol, and then stained with 0.1% crystal violet. After washing, invaded cells were counted in three random fields of each filter under a microscope. Each experiment was conducted in triplicate.

### 2.9 Immunofluorescence

Cells were seeded on a glass slide and fixed using 4% paraformaldehyde. After washed twice with PBS, these slides were then permeabilized using a 0.2% Triton X‐100 solution, and subsequently blocked with 3% BSA. Next, the slides were incubated with primary antibody. Then these slides were washed with PBS and then incubated with anti‐rabbit FITC‐conjugated secondary antibody. Nuclei were visualized with DAPI. Images were acquired by confocal laser‐scanning microscope (Olympus, LakeSuccess, NY).

### 2.10 Immunohistochemistry

Immunohistochemistry was performed using immunohistochemistry kit (Key‐GEN, Nanjing, China). After deparaffinization and rehydration, the slides were treated with 3% hydrogen peroxide and blocked with 10% goat serum. Then they were incubated with primary antibodies in PBS. After washing, secondary antibodies were added and incubated at room temperature. Streptavidin‐HRP was added. After 45 min, these slides were stained with DAB Kits and hematoxylin.

### 2.11 In vivo study

Gallbladder cancer GBC‐SD cells (1.0 × 10^6^ cells per mouse) were subcutaneously injected into 40 nude mice (Model Animal Research Center of Nanjing University, MARC). When the tumor volume reached 100 mm^3^, mice were randomly divided into 5 groups: control, SP alone, SP + L703606, SP + AKTi, SP + p65 siRNA (6 mice per group). Control group was given PBS. Mice were intratumour injection with SP (50 μg/kg) twice a week. Meanwhile, L703606 (10 mg/kg), AKTi (500 μg/kg), and P65 siRNA group (50 nmol/L per mouse) were intraperitoneal injected. The tumor volume was measured with a caliper every 3 day, using the following formula: volume = length × width^2^ × 0.5.

### 2.12 Statistical analysis

Prism (GraphPad) was used to conduct all statistical analyses. The results are expressed as means ± SEM. Statistical analysis was carried out using the independent‐samples *t*‐test or one‐way ANOVA, with *P *<* *0.05 considered to indicate a statistically significant difference.

## 3 RESULTS

### 3.1 SP and NK‐1R were highly expressed in human gallbladder carcinoma

**Figure 1:**
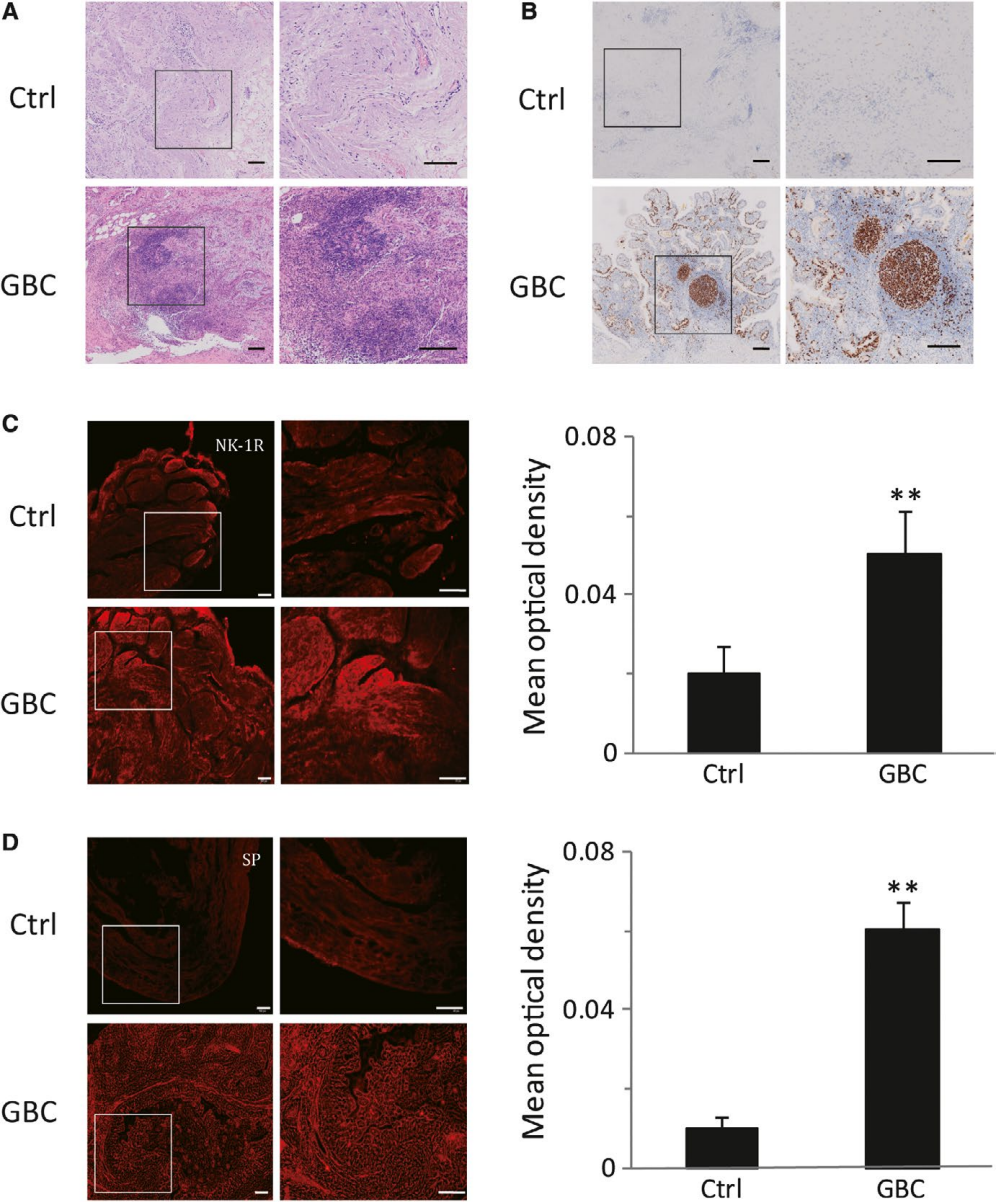
SP and NK‐1R were highly expressed in human gallbladder cancer. Serial sections of gallbladder tissue were stained with HE measured (scale bar, 100 μm) (A). The immunohistochemical stain for Ki‐67 in gallbladder samples were detected (scale bar, 100 μm) (B). Sections of human gallbladder tissue were immunostained for NK‐1R (red) (scale bar, 100 μm) (C). Expression of SP was analyzed by immunofluorescence cytochemistry (scale bar, 100 μm) (D). Data were shown as means ± SEM (Ctrl: n = 12, GBC: n = 8). Statistical analysis was performed using one‐way ANOVA coupled with a post hoc test. Significant differences were indicated as **P <* *0.01 versus Ctrl

It has been reported that SP/NK‐1R system plays an important role in a number of tumors^6^. And binding of SP to NK‐1R initiates tumor cell proliferation, which is critical for tumor invasion and metastasis. In order to determine the role of SP and NK‐1R in GBC, we carried out immunostaining in 12 human GBC samples. Histological analysis (Figure 1A), Ki67 staining (Figure 1B) and immunofluorescence results (Figure 1C) revealed that SP and NK‐1R were high expressed in the GBC tissues. On the contrary, SP and NK‐1R were poorly represented in the non‐tumor part (Figure 1D). These results demonstrated that the expression levels of SP and NK‐1R are enhanced in GBC.

### 3.2 Stimulation of NK‐1R could enhance the proliferative, migrative and invaded effects in vitro

**Figure 2:**
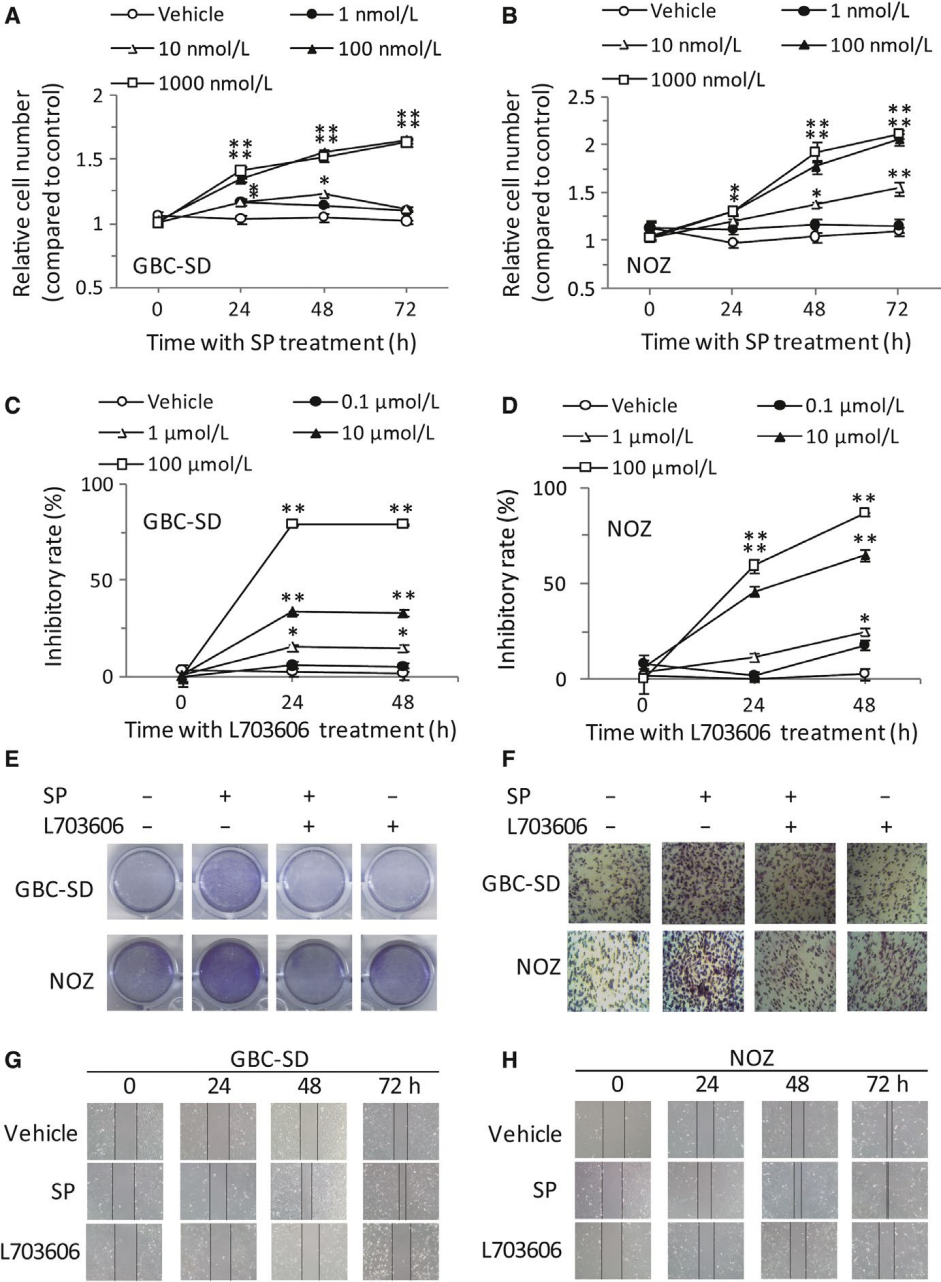
Stimulation of NK‐1R could enhance the proliferative, migrative and invaded effects in vitro. GBC‐SD cells (A) or NOZ cells (B) were seeded into 96‐well plates (6.0 × 10^3 ^cells per well) and then treated with various concentrations of SP. The cell viability was determined using MTT assay at the indicated times. GBC‐SD cells (C) or NOZ cells (D), were treated with L703606 at the indicated concentrations for different times. The cell viability was measured using MTT assay. Effects of SP/NK‐1R system on clone formation ability in GBC‐SD and NOZ cells (E). The invasion of GBC‐SD and NOZ cells were examined by transwell assay. After pretreatment with L703606, cells were stimulated with SP, and the cells that invaded across the membrane of the transwell were stained with hematoxylin and eosin (F). GBC‐SD cells were scraped with a pipette tip and then treated with SP or L703606 for different times. The migrating cells were detected using a light microscope (G). NOZ cells were scraped and migration was determined by microscope (H). Data were shown as means ± SEM (n = 6). Statistical analysis was performed using one‐way ANOVA coupled with a post hoc test. Significant differences were indicated as *P <* *0.05 versus Vehicle, **P <* *0.01 versus Vehicle

In order to determine the functional consequences of NK‐1R in GBC, GBC‐SD and NOZ cells were treated with SP at different concentrations or times and cell growth was analyzed with MTT assay. The results showed that SP could markedly increase cell proliferation in a time‐ and dose‐dependent manner. And the GBC‐SD cells growth at 100 nmol/L or 1000 nmol/L SP were more than 1.6‐fold compared to those without SP treatment at 72 hour (Figure 2A). A similar effect was observed in NOZ cells (Figure 2B). Next, we used a selective NK‐1R antagonist L703606 to block the binding of SP to NK‐1R and evaluated the growth inhibition by MTT assay. As expected, we observed a concentration‐and time‐dependent growth inhibition in vitro (*P *<* *0.01) (Figure 2C,D). Furthermore, crystal violet staining also revealed that L703606 significantly reversed the proliferative effect of SP (Figure 2E). These results suggest that the activation of NK‐1R could induce the proliferation of GBC.

To further investigate the role of NK‐1R on GBC cell migration and invasion, GBC‐SD cells was treated with SP (100 nmol/L) and L703606 (100 μmol/L) for indicated times, and then subjected to wound healing or transwell invasion assays. SP obviously promoted cells invasion, and this was markedly abolished by L703606 (Figure 2F). And further experiments showed that GBC‐SD with SP treatment gradually reduced the wound width, while the effect was opposite in the presence of NK‐1R antagonist (Figure 2G). A similar effect was observed in NOZ cells (Figure 2H).

### 3.3 Activation of NK‐1R could promote NF‐κB activation in vitro

**Figure 3:**
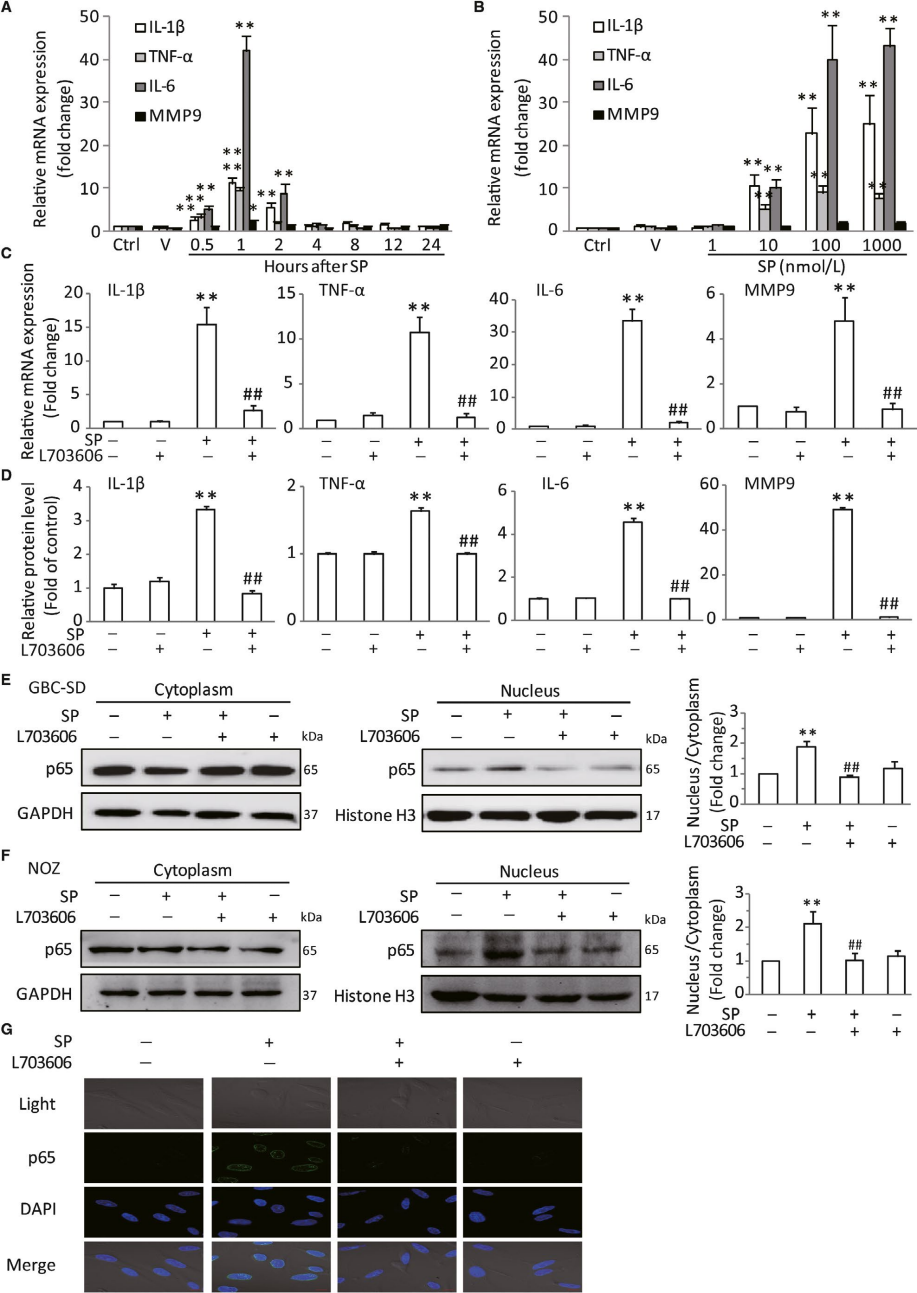
Activation of NK‐1R could promote NF‐κB activation in vitro. GBC‐SD cells were stimulated by SP with different dose for the indicated time. The mRNA production of tumor‐related cytokines IL‐6, IL‐1β, TNF‐α and MMP9 were determined by real‐time PCR, and the expression levels were normalized to β‐actin (A and B). After pretreated with L703606, the cells were stimulated with SP, the mRNA expression and protein levels in GBC‐SD cells were analysied (C and D). The NF‐κB p65 nuclear translocation in GBC‐SD and NOZ cells were assessed by western blot. Histone H3 and GAPDH were used as nuclear and cytoplasmic markers (E and F). Immunofluorescence was used to analyze NF‐κB p65 nuclear translocation (scale bar, 20 μm) (G). Data are presented as means ± SEM (n = 6). Statistical analysis was performed using one‐way ANOVA coupled with a post hoc test. Significant differences were indicated as *P <* *0.05 versus Ctrl, **P <* *0.01 versus Ctrl, ^##^ *P *<* *0.01 versus SP

**Figure 4:**
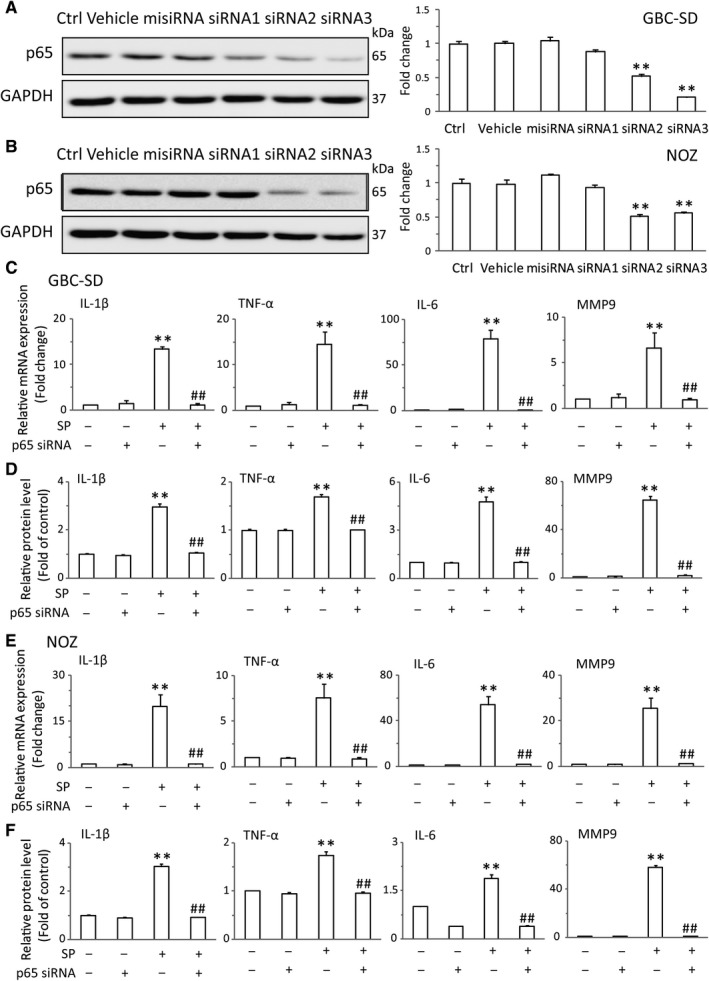
The promotive effect of NK‐1R on the NF‐κB was dependent on Akt in vitro. GBC‐SD and NOZ cells were transfected with NF‐κB p65 siRNA for 8 h, the expression of p65 was assessed by western blot (A and B). The transfected GBC‐SD cells were treated with SP, the mRNA levels of IL‐6, IL‐1β, TNF‐α and MMP9 were measured by real‐time RT‐PCR (C). And the protein levels of IL‐6, IL‐1β, TNF‐α and MMP9 were determined by ELISA (D). NOZ cells were transfected with p65 siRNA, with the treatment of SP for indicated times. Real‐time PCR analysis was performed to detect the mRNA expressions of L‐6, IL‐1β, TNF‐α and MMP‐9 (E). ELISA was performed to detect the protein levels of L‐6, IL‐1β, TNF‐α and MMP9 (F). Data are presented as means ± SEM (n = 6). Statistical analysis was performed using one‐way ANOVA coupled with a post hoc test. Significant differences were indicated as *^*^ *P *<* *0.01 versus Ctrl, ^##^ *P *<* *0.01 versus SP

**Figure 5:**
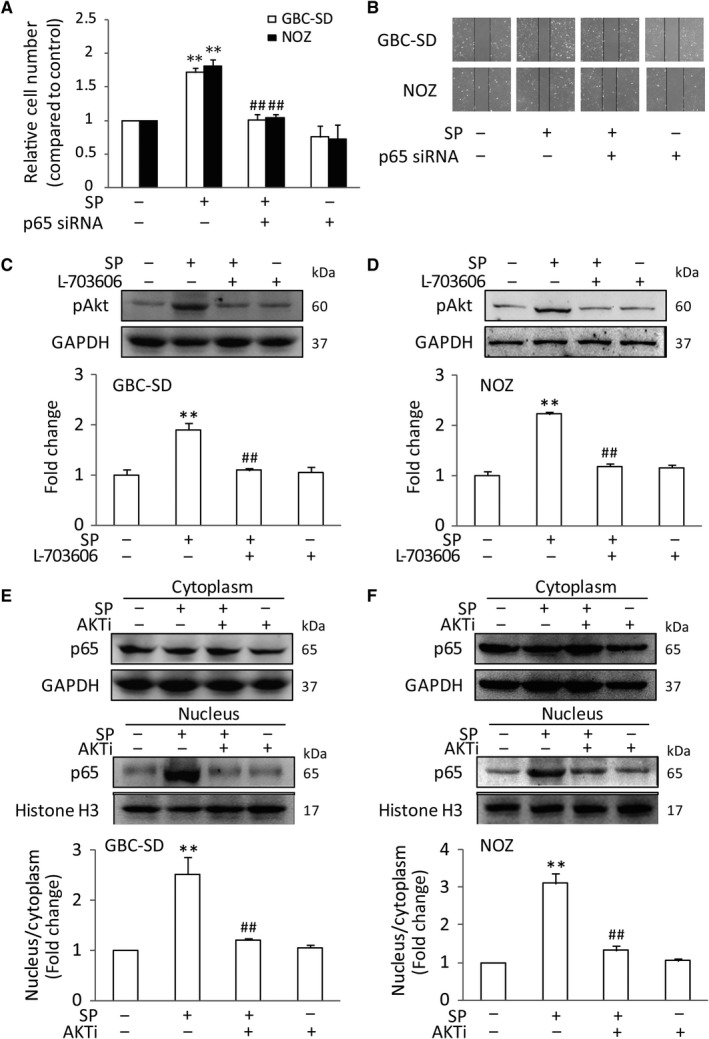
The role of the Akt on NK‐1R‐induced NF‐κB activation. GBC‐SD or NOZ cells were transfected with p65 siRNA, with the treatment of SP. The transfected cell viability was measured using MTT assay (A), The transfected cells were scraped and migration was determined by microscope (B), After pretreated with L703606, GBC‐SD and NOZ cells were stimulated with SP. The Phosphorylation of Akt was determined by western blot (C and D), GBC‐SD and NOZ cells were treated with Akt inhibitor, accompanied by SP treatment. Western blot analyses was performed to investigate nuclear translocation of NF‐κB p65. Histone H3 and GAPDH were used as nuclear and cytoplasmic markers (E and F), Data are presented as means ± SEM (n = 6). Statistical analysis was performed using one‐way ANOVA coupled with a post hoc test. Significant differences were indicated as **P <* *0.01 versus Ctrl, ^##^ *P *<* *0.01 versus SP

The tumor‐associated proinflammatory cytokines and gelatinases have been identified as the key factors in enhancing GBC tumorigenesis and progression.^13^ Among these elements, IL‐6, IL‐1β, TNF‐α and MMP9 are the pivotal cytokines and gelatinase presented in the tumor microenvironment.^26^, ^27^ Therefore, we analysed the effects of SP/NK‐1R system on these tumor‐associated mediators in GBC‐SD and NOZ cells. As expected, the mRNA levels of IL‐1β, IL‐6, TNF‐α and MMP9 were significantly increased after SP treatment. And the most obvious effects existed at 1 hour (Figure 3A,B). Moreover, Figure 3C,D revealed that L703606 could markedly attenuate the expression of these tumor‐related factors in GBC cells treated with SP. Previous studies have shown that NF‐κB is a critical regulator of these cytokines and gelatinase. Thus, we sought to investigate whether NK‐1R could affect NF‐κB pathway. Consistent with previous researches, stimulation of the GBC‐SD cells with SP obviously induced the nuclear translocation of NF‐κB p65. Meanwhile, NK‐1R antagonist effectively attenuated the NF‐κB activity, as evidenced by the down‐regulating nuclear expression of NF‐κB p65 (Figure 3E). And the similar results were observed in NOZ cells (Figure 3F). Moreover, immunofluorescent also revealed that pre‐treatment with L703606 remarkable abolished SP‐induced p65 nuclear translocation in GBC‐SD cells (Figure 3G). To test whether the effect of NK‐1R on the expressions of cytokines and gelatinase was mediated by p65, siRNA targeting p65 was used to suppress endogenous p65 expression. As shown in Figure 4A,B, siRNA 2 and siRNA 3, but not control siRNA, could markedly inhibit the p65 protein expression. Knockdown of p65 in GBC‐SD significantly decreased the SP‐induced elevated mRNA and protein levels of IL‐1β, IL‐6, TNF‐α and MMP9 (Figure 4C,D). And the p65 siRNA also resulted in a sharp decrease of their elevated expressions in NOZ (Figure 4E,F). Next, we investigated the effects of p65 siRNA on the proliferation and invasion in GBC cells. As shown in Figure 5A,B, p65 interference strongly inhibited cell proliferation and invasion, compared with the normal group in both cell lines. These results demonstrated that the NK‐1R‐induced NF‐κB activation promotes the migration and invasion of GBC cells.

### 3.4 The promotive effect of NK‐1R on the NF‐κB was dependent on Akt in vitro

**Figure 6:**
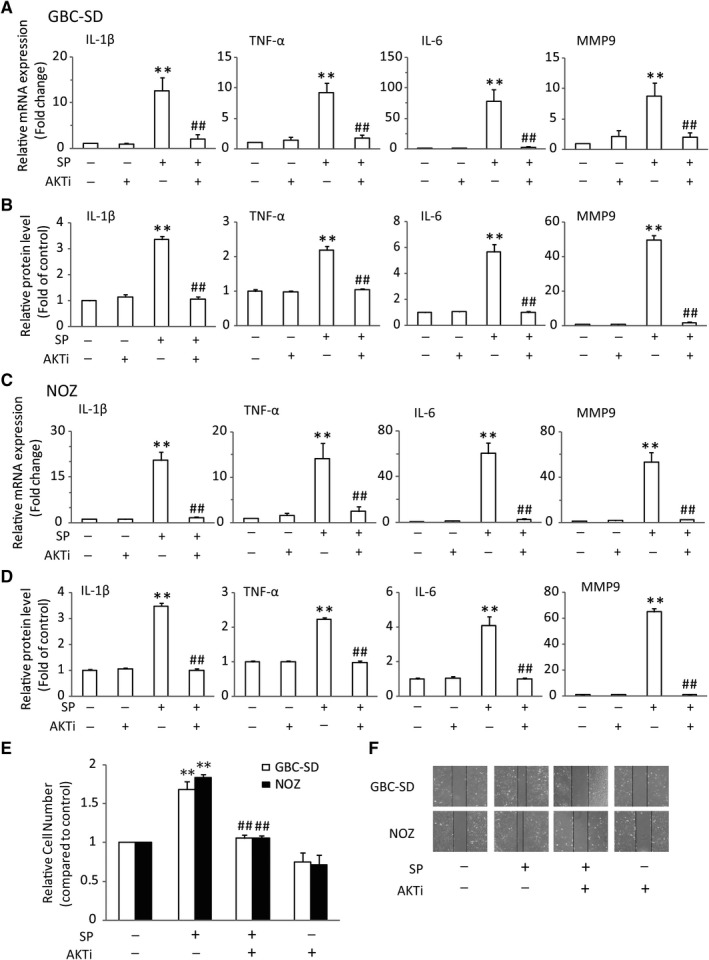
Activation of NK‐1R induced the expression of NF‐κB‐associated cytokines, and proliferative, migrative in GBC cells through up‐regulating the Akt pathway. GBC‐SD cells were treated with Akt inhibitor, accompanied by SP treatment. The mRNA levels of IL‐6, IL‐1β, TNF‐α and MMP9 were measured by real‐time RT‐PCR. And the protein levels of IL‐6, IL‐1β, TNF‐α and MMP9 were determined by ELISA (A and B), After pretreated with L703606, NOZ cells were stimulated with SP. Real‐time RT‐PCR was performed to detect the mRNA levels of IL‐6, IL‐1β, TNF‐α and MMP9. And ELISA was performed to detect the secretion of IL‐6, IL‐1β, TNF‐α and MMP9 (C and D), The cell viability was measured using MTT assay (E), The cells were scraped and migration was determined by microscope (F), Data are presented as means ± SEM (n = 6). Statistical analysis was performed using one‐way ANOVA coupled with a post hoc test. Significant differences were indicated as **P <* *0.01 versus Ctrl, ^##^ *P *<* *0.01 versus SP

It is noted that Akt could activate the NF‐κB pathway and up‐regulate the transcription of pro‐survival genes, suggesting a mechanistic link between Akt and NF‐κB. Therefore, we hypothesized that the Akt might involve in the effect of SP/NK‐1R system on NF‐κB signaling. The effects of SP and L703606 on the phosphorylation of Akt (pAkt) were tested. Indeed, we found that SP could significantly trigger the occurrence of Akt activation in GBC‐SD and NOZ cells. However, when NK‐1R antagonist was given to cells followed by SP stimulation, the phosphorylation of Akt was markedly repressed (Figure 5C,D). To further test if the increased Akt activity is responsible for the p65 nuclear translocation, Akt activity was reduced with a pharmacological Akt inhibitor, AKTi. The results showed that AKTi could significantly relieve the p65 nuclear expression in GBC‐SD and NOZ cells after SP treatment (Figure 5E,F). Consistent with this, SP had little effect on the mRNA levels and protein secretions of IL‐1β, TNF‐α, IL‐6 and MMP9 after AKTi intervention in GBC cells (Figure 6A‐D). What's more, the inhibition of Akt function in GBC cells significantly attenuated SP‐induced cell proliferation and invasion, compared with SP‐treated cells. The similar results were observed in NOZ cells (Figure 6E,F). Taken together, these data indicated that Akt play an important role in the NK‐1R‐induced NF‐κB activation, as well as in cell migration and invasion.

### 3.5 NK‐1R/Akt/NF‐κB signaling pathways was activated in human gallbladder cancer

**Figure 7:**
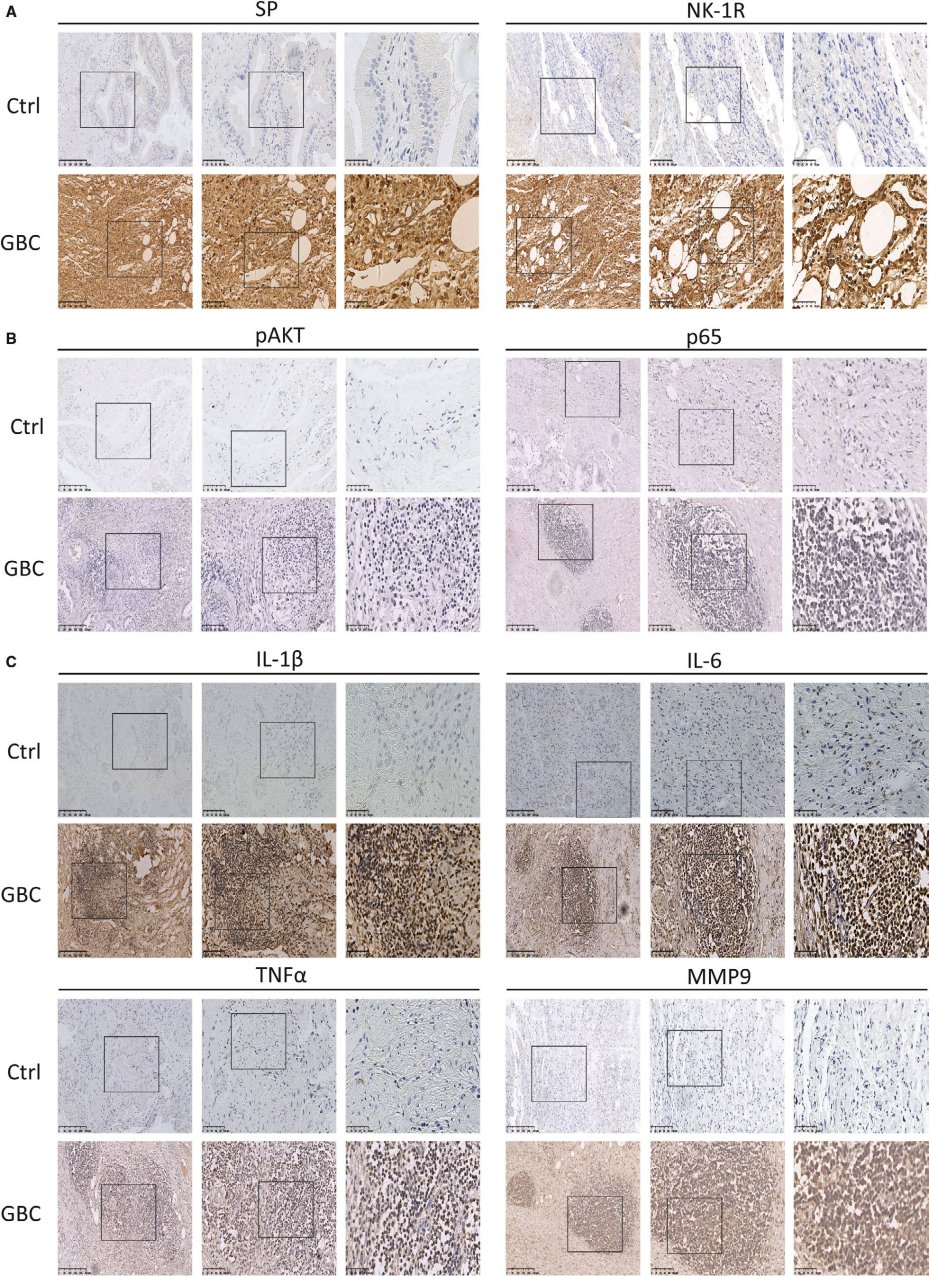
NK‐1R/Akt/NF‐κB signaling pathways is activated in human gallbladder cancer. (A, B and C), The expressions of SP, NK‐1R, pAkt, p65, IL‐6, IL‐1β, TNF‐α and MMP9 in tumor tissues were detected by immunohistochemistry (Ctrl: n = 12, GBC: n = 8)

Prior work implicated that NK‐1R/Akt/NF‐κB pathway was critical in GBC cell growth, invasion and migration in vitro. We further used immunohistochemistry to assess the role of this pathway in vivo. As shown in Figure 7, consistent with previous data, immunohistochemical staining revealed a high expression of SP and NK‐1R in human GBC tissues, which was significantly higher than those in the non‐tumor ones (Figure 7A). Moreover, we analyzed the Akt level and found that the activity of Akt was increased in GBC. Furthermore, NF‐κB p65 was obviously over‐expressed in the GBC samples (Figure 7B). And the expression of IL‐6, IL‐1β, TNF‐α and MMP9 in tumor samples were also markedly up‐regulated compared with non‐tumor parts (Figure 7C). These results suggested that the protein levels in NK‐1R/Akt/NF‐κB pathway were increased and the downstream targets, such as IL‐6 and MMP9, were also triggered.

### 3.6 Suppression of NK‐1R/Akt/NF‐κB signaling pathway inhibited SP‐induced GBC cell growth in vivo

**Figure 8:**
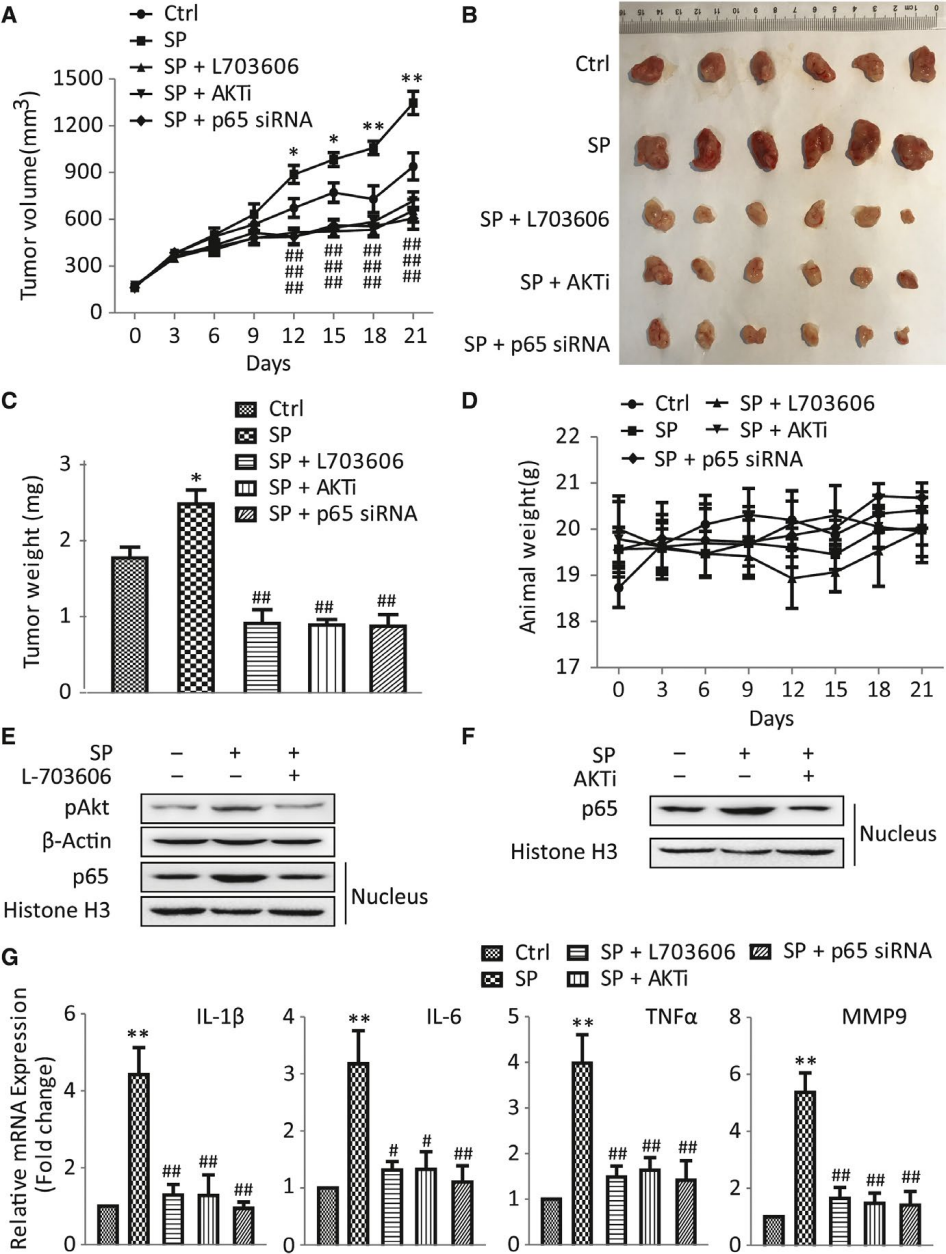
Suppression of NK‐1R/Akt/NF‐κB signaling pathways inhibited SP‐induced GBC cell growth in vivo. A, Tumor size of nude mice was measured by vernier caliper at the end of the treatment time in all groups. B and C, Weight of tumor obtained from tumor‐bearing mice is shown. D, Weight changes of nude mice in five separate groups were measured for 21 days. E and F, The phosphorylation of Akt and nuclear p65 of tumors were determined by western blot. G, Real‐time RT‐PCR was performed to detect the mRNA levels of IL‐6, IL‐1β, TNF‐α and MMP9 in tumors

To determine the effect of NK‐1R/Akt/NF‐κB signaling pathway in vivo, nude mice were xenografted with the GBC‐SD cells. At day 12, tumors injected with SP alone appeared larger than the control group. In comparison, L703606, AKTi and p65 siRNA resulted in a significantly inhibitory effects on SP‐induced tumor growth throughout the entire treatment period (Figure 8A). At 21 days, the mice were sacrificed and tumor weight was measured. Results showed the same conclusion. SP group exhibited increased tumor weight, while there was a markable reduction after L703606, AKTi or p65 siRNA treatment (Figure 8B,C). Consistent with previous results, SP greatly enhanced protein level of pAkt and nuclear translocation of NF‐κB p65 in vivo. Meanwhile, the mRNA expressions of IL‐1β, IL‐6, TNF‐α and MMP9 were also increased. However, L703606 significantly attenuated this phenomenon. Furthermore, combined with SP, AKTi obviously led to a sharp decrease of IL‐1β, IL‐6, TNF‐α and MMP9, and treatment with p65 siRNA had the similar effects. Moreover, it was found that AKTi markedly decreased SP‐induced NF‐κB activation (Figure 8E‐G). Taken together, these results strongly suggested that SP could promote the GBC growth through the NK‐1R/Akt/NF‐κB pathway.

## 4 DISCUSSION

Over the past decades, the function of peptides and their receptors in tumors have attracted growing interest, and research efforts show that these peptides and receptors can influence cancer progress. One such target could be SP/NK‐1R system, since it is known that NK‐1R can act as a mitogen on several human cancers.^6^, ^28^, ^29^, ^30^ Currently, it is still unknown whether NK‐1R exists the same effect in GBC. In this report, our results showed that SP and NK‐1R were expressed at high protein levels in GBC tumor samples. Consistent with previous researches,^6^, ^31^ the activation of NK‐1R dose‐dependently induced the proliferation and invasion of GBC cells, and these effects could be blocked by NK‐1R antagonist. Notably, we also observed that NK‐1R could significantly increase the activity of NF‐κB throuth up‐regulating Akt. And Akt inhibitor could decrease the nuclear translocation of NF‐κB p65 induced by SP, which reduced the high‐production of proinflammatory cytokines and gelatinase. Furthermore, we also detected the increased protein levels in NK‐1R/Akt/NF‐κB signaling in human GBC tissues. Moreover, results from xenografts in nude mice suggested that the inhibition of NK‐1R/Akt/NF‐κB pathway could cancel the proliferative effect of SP on GBC. These present data suggested that NK‐1R could drive tumor growth through the Akt/NF‐κB in GBC.

It is known that the biological action of SP is mainly mediated by the tachykinin receptor, NK‐1R, which belongs to the G protein‐coupled receptors (GPCRs). So far, there were only three types of tachykinin receptors have been identified, designated NK‐1, NK‐2 and NK‐3.^32^ Earlier studies demonstrated that the biological activities of NK‐1R are encoded by the C‐terminal sequence of tachykinins, for which SP is a more potent agonist than other peptides. Moreover, the activation of NK‐1R by SP can induce the hydrolysis of membrane phosphoinositides, and then lead to the formation of intracellular inositol 1,4,5‐triphosphate and diacyl‐glycerol.^33^, ^34^ The transient increasing of these messages will trigger a cascade of protein phosphorylation/dephosphorylation reactions, which are critical for gene expression and cell function.^30^ This interaction occurs in various physiologic and pathophysiologic responses which are mediated by NK‐1R, such as sensory perception, emesis, neuronal degeneration, inflammation, wound healing, pain, anxiety, and the regulation of the cardiovascular system.^6^ In addition, NK‐1R has also been reported to be over‐expressed in many human cancer cells, as well as in primary tumors. For example, up‐regulation of NK‐1R is localized in human melanoma, glioma, neuroblastoma, leukemia, lung cancer, gastric and colon adenocarcinomas.^9^, ^28^ It is also known that SP, via NK‐1R, induces tumor cell proliferation and metastasis in a concentration‐dependent manner.^7^ Moreover, researchers find that the increased expression of SP and NK‐1R in tumor tissues are related with the degree of malignancy of cancers.^6^ The immunohistochemistry also revealed a high density of SP and NK‐1R associated with tumor node metastasis in colorectal cancer.^10^ And SP/NK‐1R system have also been observed to play a crucial role in the progression of endometrial carcinoma by promoting tumor metastasis.^11^ By contrast, NK‐1R antagonists elicits growth inhibition in a large number of tumor cell types by inducing apoptosis.^35^ R. Coveñas et al. report that NK‐1R antagonist treatment can induce gastrointestinal cancer cells apoptosis and inhibit tumor proliferation.^36^ Moreover, NK‐1R antagonists hypothetically alleviate the ailments which go along with cancer conventional therapy. Thus, NK‐1R antagonist can be represented as a promising antitumor drug for the treatment against the NK‐1 receptor in cancer. Our results are in agreement with previous works reporting the function of NK‐1R in human cancers.^12^, ^29^, ^31^ In GBC samples, the protein level of SP and NK‐1R markedly elevated (Figure 1), which indicates the connection between NK‐1R and cancer. We also demonstrated that SP dose‐ and time‐dependently promoted proliferation, migration and invasion of GBC cells through triggering NK‐1R activity in vitro. However, these results were remarkably abolished after L‐703606 treatment. In addition, our finding also revealed that SP could significantly promote of GBC growth in vivo, while NK‐1R antagonist could cancel the effect.

When NK‐1R are activated by SP, this can induce the release of several second messengers which trigger a broad array of effector mechanisms involved in the regulation of cancer cell function. For example, it has been widely accepted that the stimulation of the NK‐1R by SP can markedly increase the activation of NF‐κB, and the over‐activation of NF‐κB positively regulates the transcription factors that favour tumorigenesis.^13^, ^37^ In our study, SP could induced the activation of NF‐κB and its downstream targets**,** including IL‐1β, TNF‐α, IL‐6 and MMP9. However, NK‐1R antagonist dramatically inhibited the nuclear translocation of NF‐κB p65 and other cytokine production. Furthermore, we use p65 siRNA to evaluate the role of NF‐κB on SP/NK‐1R system in vitro and in vivo. And the results indicated that NF‐κB played a key role in SP/NK‐1R‐induced proliferation, migration, invasion and cytokines secretion in GBC.

In addition, previous studies have demonstrated that the activation of Akt can significantly trigger NF‐κB activation, which promotes the expression of downstream proinflammatory cytokines and gelatinase, and then induces a series of aggressive phenotypes.^38^, ^39^ In human glioblastoma cells, the NK‐1R agonist can markedly increase the phosphorylation and activity of Akt whereas treatment with the NK‐1R antagonist leads to reducing the basal Akt kinase activity, which is involved in the apoptosis.^6^, ^40^ Therefore, we focused our attention on the phosphorylation of Akt, which may be a key factor in the SP/NK‐1R system. Our results showed that NK‐1R agonist markedly promoted the activation of Akt in GBC. And the results with the Akt inhibitor treatment demonstrated that Akt, as an up‐steam regulator, played an important role in NK‐1R‐induced NF‐κB activation. In addition, we evaluated the effects of Akt inhibitor in vivo. Our study shows that Akt inhibitor prevented the SP‐induced GBC growth. We also find that both the translocation of NF‐κB p65 and the downstream cytokine production were significantly inhibited by Akt inhibitor. Furthermore, immunohistochemical staining revealed that SP, NK‐1R, pAkt, NF‐κB p65 and the other cytokines were highly expressed in tumor tissues. Taken together, these results indicated that SP obviously induced cancer cell proliferation, migration and invasion through regulating the NK‐1R/Akt/NF‐κB axis.

In conclusion, we provide evidence that NK‐1R and its agonist SP are highly expressed in GBC tissues. SP could induce the proliferation, metastasis and invasion of GBC cells and promote tumor growth in vivo, which is regulated by NK‐1R/Akt/NF‐κB pathway. The results extend our understanding on the molecular mechanisms of SP/NK‐1R on the tumorogenesis activity in GBC. Therefore, this study suggested that the SP/NK‐1R complex might act as a novel therapeutic target in human GBC and warrant further studies in order to explore anticancer strategies against GBC.

## CONFLICT OF INTEREST

The authors declare that they have no conflict of interests.

## AUTHOR CONTRIBUTION

LM and XTD designed research; XTD, SMT, PYW, QPL, XXG, BMX, and HSW performed research; XTD, SMT, and LM analyzed data; XTD and LM wrote the paper.
